# RNA-binding protein GLD-1/quaking genetically interacts with the *mir-35* and *the let-*7 miRNA pathways in *Caenorhabditis elegans*

**DOI:** 10.1098/rsob.130151

**Published:** 2013-11

**Authors:** Alper Akay, Ashley Craig, Nicolas Lehrbach, Mark Larance, Ehsan Pourkarimi, Jane E. Wright, Angus Lamond, Eric Miska, Anton Gartner

**Affiliations:** 1 Centre for Gene Regulation and Expression, University of Dundee, Dundee DD1 5EH, UK; 2 Wellcome Trust Cancer Research UK Gurdon Institute, University of Cambridge, Cambridge CB2 1QN, UK; 3 Friedrich Miescher Institute for Biomedical Research, Basel 4002, Switzerland

**Keywords:** *Caenorhabditis elegans*, miRNA, gld-1, let-7, SILAC

## Abstract

Messenger RNA translation is regulated by RNA-binding proteins and small non-coding RNAs called microRNAs. Even though we know the majority of RNA-binding proteins and microRNAs that regulate messenger RNA expression, evidence of interactions between the two remain elusive. The role of the RNA-binding protein GLD-1 as a translational repressor is well studied during *Caenorhabditis elegans* germline development and maintenance. Possible functions of GLD-1 during somatic development and the mechanism of how GLD-1 acts as a translational repressor are not known. Its human homologue, quaking (QKI), is essential for embryonic development. Here, we report that the RNA-binding protein GLD-1 in *C. elegans* affects multiple microRNA pathways and interacts with proteins required for microRNA function. Using genome-wide RNAi screening, we found that *nhl-2* and *vig-1*, two known modulators of miRNA function, genetically interact with GLD-1. *gld-1* mutations enhance multiple phenotypes conferred by *mir-35* and *let-7* family mutants during somatic development. We used stable isotope labelling with amino acids in cell culture to globally analyse the changes in the proteome conferred by *let-7* and *gld-1* during animal development. We identified the histone mRNA-binding protein CDL-1 to be, in part, responsible for the phenotypes observed in *let-7* and *gld-1* mutants. The link between GLD-1 and miRNA-mediated gene regulation is further supported by its biochemical interaction with ALG-1, CGH-1 and PAB-1, proteins implicated in miRNA regulation. Overall, we have uncovered genetic and biochemical interactions between GLD-1 and miRNA pathways.

## 2. Introduction

microRNAs (miRNAs) are approximately 21 nucleotide-long endogenous regulatory RNAs that mediate translational regulation through binding to the 3′UTR of target mRNAs. Over the past decade, they have been implicated in many biological processes and are now considered to be major modulators of gene expression [1,2]. After biogenesis, miRNAs and Argonaute proteins (AGO; ALG-1/ALG-2 in *C. elegans*) form the miRNA-induced silencing complex (miRISC) together with GW182 proteins (AIN-1/AIN-2 in *C. elegans*) [3]. VIG-1 is thought to function in the miRNA pathway and was identified as an interactor of miRNA/Argonaute complexes by co-immunoprecipitation [4]. Mammalian TRIM32 and *C. elegans* NHL-2 also interact with AGO and promote miRNA activity [5,6].

GLD-1 is a member of a highly conserved RNA-binding protein family, characterized by the signal transduction and activation of RNA (STAR) domain [7]. GLD-1 affects *C. elegans* germline development and maintenance by translational repression of a variety of target proteins [8–14]. A key role for GLD-1 in modulating DNA damage-induced germline apoptosis was uncovered via the hypomorphic *gld-1*(*op236*) allele [13]. In *gld-1*(*op236*) mutants, GLD-1 is unable to bind the 3′UTR of *cep-1*/p53, whereas translational repression of GLD-1 targets mediating developmental regulation is unaffected. *gld-1*(*op236*) is unique among *gld-1* alleles in showing no overt defect in germ cell development at the permissive temperature. However, at the restrictive temperature, *gld-1*(*op236*) animals are sterile, and undifferentiated germ cells accumulate [13]. The mammalian orthologue of GLD-1 is quaking/QKI, which functions in translational regulation during neuronal development [15,16]. GLD-1 and QKI are functionally conserved, and ectopically expressed QKI in worms recognizes GLD-1 target sequences [17]. Although GLD-1 biochemically interacts with AIN-2, the functional consequences of this interaction have not yet been determined [18]. Lastly, miRNA-related functions of *gld-1* have not been documented by mutational analysis, and a *gld-1* phenotype affecting somatic development of animals has not been reported.

Deleting the vast majority of known *C. elegans* miRNAs individually does not result in obvious overt phenotypes [19]. Phenotypes tend to arise when several members of a miRNA family are deleted [20]. Alternatively, mutating miRNA pathway genes also generate sensitized system that helps us to unravel miRNA function [21]. Such synthetic phenotypes point towards the existence of extensive redundancy in miRNA-mediated gene regulation. *Caenorhabditis elegans* genetics allows for using ‘sensitized’ genetic backgrounds to study subtle phenotypes associated with redundant mechanisms of miRNA-mediated gene regulation.

Initially aiming to identify genes required for GLD-1-mediated translational regulation, we performed a genome-wide RNAi screen for enhancers of the *gld-1(op236)* hypomorphic allele. This screen identified *vig-1* and *nhl-2*, both of which are modulators of miRNA function, thus suggesting that GLD-1 might affect general miRNA-mediated gene regulation. We indeed found that *gld-1* enhances multiple *let-7* and *mir-35* family miRNA phenotypes affecting somatic development. Using stable isotope labelling with amino acids in cell culture (SILAC)-based proteomics, we show that the upregulation of the histone mRNA-binding protein CDL-1 is partially responsible for the genetic interactions between GLD-1 and let-7 miRNA. A role for GLD-1 in miRNA-mediated gene regulation is further supported by the interaction of GLD-1 with ALG-1, CGH-1 and PAB-1, proteins previously implicated in miRNA-mediated gene regulation.

## 3. Material and methods

### 3.1. Strains and animal handling

Strains used in this paper were **TG34** *gld-1*(*op236*)*I*, **TG2209** *vig-1*(*ok2536*)*II*, **TG2129** *gld-1*(*op236*)*I; vig-1*(*ok2536*)*II*, **TG1725** *nhl-2*(*ok818*)*III*, **TG2130** gld-1(op236)I; nhl-2(ok818)III, **MT14119** *nDf50II*, **TG2133** *gld-1*(*op236*)*I; nDf50II,* **GR1432** *let-7*(*mg279*)*X*, **TG1684** *gld-1*(*op236*)*I; let-7*(*mg279*)*X*, **VT1142** *nDf51V; mir-84 (n4037)X; ctIs39*, **TG2134** *gld-1*(*op236*)*I; nDf51V; mir-84* (*n4037*)*X; ctIs39*, **SU93** *jcIs1IV, TG2017 gld-1*(*op236*)*I; jcIs1IV*, **TG2018** *jcIs1IV; let-7*(*mg279*)*I*, **TG2019** *gld-1*(*op236*)*I; jcIs1IV; let-7*(*mg279*)*X*, **MT2124** *let-60*(*n1046*)*IV*, **TG2135** *gld-1*(*op236*)*I; let-60*(*n1046*)*IV*, **SD551** *let-60*(*ga89*)*IV,* **TG2136** *gld-1*(*op236*)*I; let-60*(*ga89*)*IV*, **TG1828** *maIs105V*, **TG1825** *gld-1*(*op236*)*I; maIs105V*, **TG1826** *maIs105V; let-7*(*mg279*)*X*, **TG1827** *gld-1*(*op236*)*I; maIs105V; let-7*(*mg279*)*X*, **TG2137** *gld-1*(*op236*)/*hT2 I; maIs105V; let-7*(*mg279*)*X*, **TG2138** *gld-1*(*op236*)/*gld-1*(*q485*)*I; maIs105V; let-7*(*mg279*)*X*, **TG2131** *gld-1*(*op236*)*I; vig-1*(*ok2536*)*II; maIs105V*, **SX493** *Pcol-10::GFP::lin-41–3′UTR*(*mjIs32*) *II*, **TG2039** *Pcol-10::GLD-1::mCherry::gld-1–3′UTR*(*gtEx2039*), **TG2139** *Pcol-10::GFP::lin-41–3′UTR*(*mjIs32*) *II; Pcol-10::GLD-1::mCherry::gld-1–3′UTR*(*gtEx2039*), **TG2041** *Pgld-1::mCherry-H2B::gld-1–3′UTR*(*gtEx2041*), **SX1257** *Pcol-10::GFP::lin-41–3′UTR*(*mjIs32*) *II; Pcol-10::mCherry::unc-54–3′UTR* (*mjIs117*) *IV,* **TG2212** *Pcol-10::GFP::lin-41–3′UTR*(*mjIs32*) *II; Pcol-10::mCherry::unc-54–3′UTR* (*mjIs117*) *IV; let-7*(*mg279*), **TG2213** *gld-1(op236) I; Pcol-10::GFP::lin-41–3′UTR*(*mjIs32*) *II; Pcol-10::mCherry::unc-54–3′UTR* (*mjIs117*) *IV; let-7*(*mg279*), **TG1769** *gld-1*(*op236*) *I; Pcol-10::GFP::lin-41–3′UTR*(*mjIs32*) *II; Pcol-10::mCherry::unc-54–3′UTR* (*mjIs117*) *IV*, **OZIS2** gld-1::GFP::FLAG; gld-1(q485) (ozIs2), **SX2695** gld-1(op236) I; col-10::GFP::lin-41 deletion (mjSi35) II; unc-119(ed3) III; let-7(mg279) X, **SX2696** gld-1(op236) I; col-10::GFP::lin-41 deletion (mjSi35) II; unc-119(ed3) III, **SX2697** col-10::GFP::lin-41 deletion (mjSi35) II; unc-119(ed3) III; let-7(mg279) X, **SX2279** col-10::GFP::lin-41 deletion (mjSi35) II; unc-119(ed3) III.

*Caenorhabditis elegans* larvae were grown on *Escherichia coli* strain OP50 at 20°C unless otherwise stated. *let-60*(*ga89*) mutants were grown at 20°C and their progeny was shifted to 25°C at the L1 larval stage and scored at adult stage for the presence of multi-vulva phenotype. Microscopic analysis of the animals was carried out by anaesthetizing with levamisole and observed using a Zeiss axioscope.

### 3.2. RNAi in *Caenorhabditis elegans*

We used the whole-genome RNAi library generated by Ahringer Laboratory [22,23], and a modified version of the RNAi feeding protocol described in [24]. RNAi-expressing bacteria were grown from frozen stocks overnight at 37°C in LB medium containing 50 μg ml^−1^ ampicillin and 10 μg ml^−1^ tetracycline in 96-well plates. A fresh culture seeded from the overnight culture was incubated at 37°C in 96-deep-well plates until OD_600 nm_ was 0.6–1 and then induced with 1 mM IPTG for 2 h at 20°C. L1 larvae were dispensed into 96-well plates (10 worms well^−1^) in 100 μl M9 medium supplemented with 50 μg ml^−1^ ampicillin, 10 μg ml^−1^ tetracycline, 10 μg ml^−1^ cholesterol, 0.1 μg ml^−1^ fungizone and 1 mM IPTG. Induced bacteria (50 μl) were also dispensed into each well, and worms were grown at 20°C with constant shaking at 180 r.p.m. The presence of progeny was scored after 4–5 days. To validate candidates from the RNAi screen, 500–1000 L1 larva were treated with RNAi in 50 ml falcon tubes. Volumes of M9 medium and bacterial culture were scaled up accordingly. RNAi of GLD-1 protein interactors and *glp-1* was performed in a similar manner in 50 ml falcon tubes, and worms were transferred to plates seeded with the RNAi bacteria at L2–L3 stage. Number of assayed animals is presented on related figures.

### 3.3. Generation of transgenic lines

The pgld-1::mCherryHis::gld-1–3′UTR (GA_AA006, *gtEx2041*) construct was generated by cloning the *gld-1* promoter (amplified using primers 5′-atatatatggcgcgccTTCGAT TCATTTTATAAAACTCTG-3′ and 3′-atatatatgcggccgcTCTTCGATGGTTAACCTGTAAG-5′ from genomic DNA) using *Asc*I and *Not*I enzymes. mCherryHis was amplified using primers 5′-tatatatagcggccgcATGGTCTCAAAGGGTGAAG-3′ and 3′-atatatatggccggccTTACTTGCTGGAAGTGTACTTG-5′, and digested with *Not*I and *Fse*I. The *gld-1* 3′UTR was amplified using primers 5′- atatatatttaattaaAAAGTTCACATT TATAACTCACACTC-3′ and 3′-atatatatgggcccTTGAATAAAAACTATTTTTTATTATTTTATCTC-5′ from genomic DNA and digested with *Pac*I and *Apa*I. All fragments were cloned into a vector containing the *unc-119*(*+*) selectable marker [25]. The resulting construct was injected into worm gonads at 100 ng μl^−1^ concentration. pcol-10::GLD-1::mCherry::gld-13′UTR (GA_AA010, *gtEx2039*) construct was generated by PCR amplification of the *col-10* promoter using primers 5′-atatatatggcgcgccGGTCGTGAATTCCCTTACGA-3′ and 3′- atatatatgcggccgcGACTGAAAGCCAGGTACCTTATTC-5′ from genomic DNA and digesting with *Asc*I and *Not*I. The *gld-1* coding region was amplified from genomic DNA using primers 5′-atatatatgcggccgcATGCCGTCGTGCACCACTC-3′ and 3′- atatatatggccggccCGAAAGAGGTGTTGTTGACTG-5′ and digested with *Not*I and *Fse*I. MCherry was amplified using primers 5′-atatatatggccggccATGGTCTCAAAGGGTGAAG-3′ and 3′-atatatatttaattaaTTACTTATACAATTCATCCATGCCAC-5′ and digested with *Fse*I and *Pac*I. The *gld-1* 3′UTR was amplified as described above. DNA fragments were cloned into same backbone as above, and transgenic lines were generated by particle bombardment (PDS-100/He biolistic particle delivery system, Bio-Rad; [26]). mjIs32, mjIs117, mjSi35 constructs were generated using the *col-10* promoter, GFP and mCherry coding sequences and the *lin-41* and *unc-54* 3′UTRs as previously described [27,28] using transposon-mediated homologous recombination [29]. Lin-41 deletion 3′UTR was constructed using the primers 5′-CTGGGGGAATTCcaaaattcgttcgattttttggaaaaacctac-3′ and 5′-GAATTTTGGAATTCccccagtgttcatttaagctcccca-3′.

### 3.4. Immunoprecipitation

Anti-GLD-1 antibodies generated in our laboratory were used for GLD-1 immunoprecipitation [30]. Frozen N_2_ wild-type worm pellets (approx. 300 µl) were thawed in 2× volume lysis buffer (10 mM Tris–HCl, pH 7.5, 150 mM NaCl, 0.5 mM EDTA, 0.5% NP40, Roche mini complete protease inhibitor cocktail, 1 mM PMSF), lysed by bead beating (3 × 20 s, with 20 s intervals) with 300 µl 0.7 mm zirconia beads at 4°C. Lysates were incubated on ice for 30 min and then clarified at 13 000 r.p.m. for 10 min, 4°C. Lysate (2 mg) pre-cleared with protein G sepharose beads was incubated with 1 µg of rabbit anti-GLD-1 antibody for 1 h at 4°C or no-Ab beads, then added to 50 µl protein G sepharose beads and incubated for a further 1 h at 4°C. Beads were washed twice with wash buffer (10 mM Tris–HCl pH 7.5, 300 mM NaCl, 0.5 mM EDTA, 1× protease inhibitor cocktail, 1 mM PMSF), then protein complexes were eluted with 20 µl SDS sample buffer, separated on SDS gels, silver-stained and analysed by mass spectrometry. Immunoprecipitation of GFP–GLD-1 complexes from *gld-1* (*q485*); ozIs2 [gld-1::gfp::FLAG] [13] was carried out as described previously [31].

### 3.5. Northern blotting

Total RNA was extracted using Qiazol (Qiagen) with 300 µl of 0.7 mm zirconia beads at 4°C from synchronized adult-stage worms and separated on 15% polyacrylamide gels (SequaFlowGel). Gels were blotted onto Hybond-N (Amersham) membrane using a semi-dry electro blotter, and chemical cross-linking was carried out using l-ethyl-3-3-dimethylaminopropyl carbodiimide (EDC) as previously described [32]. Blots were probed with labelled let-7 RNA and U6 snRNA DNA oligonucleotides as previously described [33].

### 3.6. SILAC in nematodes

SILAC growth conditions, subcellular fractionations, peptide preparations and mass spectrometry were carried out as described in [34–36]. The number of proteins detectable by mass spectrometry was increased by subcellular fractionation [34]. For determining B to A and C to A ratios, we considered proteins that were only detected in one experiment if a minimum of two peptides were detected (the detection of two or more independent peptides all passing though MAXQUANT adding to the statistical significance [37]). We also considered proteins detected in two independent experiments but excluded those where the standard error of the mean based on multiple detected peptides from the same protein was bigger than the mean. In the B to A comparison, 2708 proteins passed those criteria, whereas 2927 passed in the C to A comparison. We focused on the 2179 proteins that were reliably detected in both datasets (see electronic supplementary material, table S1).

## 4. Results

### 4.1. A genetic screen identifies *vig-1* and *nhl-2* as enhancers of *gld-1*(*op236*)

**Figure 1.**
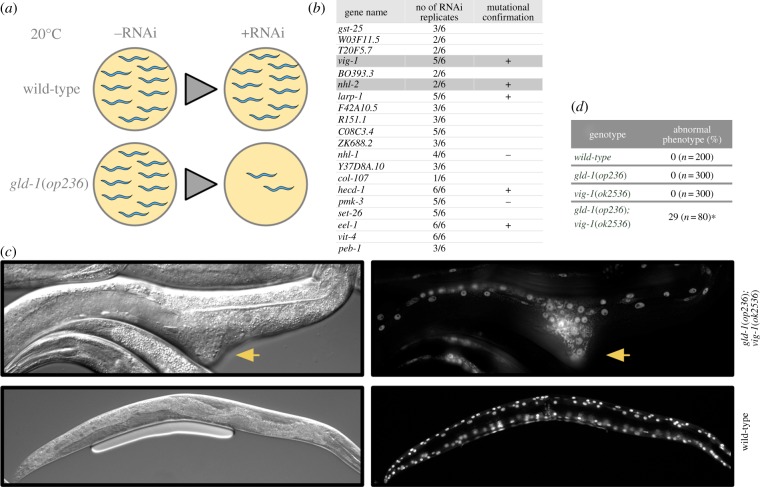
Whole genome RNAi screen identifies enhancers of *gld-1*(op236). (*a*) Schematic of the RNAi screen. (*b*) List of candidate genes with various levels of reproducibility indicated. Owing to variations in the effectiveness of RNAi, further validation was carried out by the analysis of mutants (genes marked with + or −) and five out of seven genes validated the RNAi results when tested with mutants (+). (*c,d*) Somatic defects in *gld-1*(*op236*)*; vig-1*(*ok2536*)*.* 29% of the surviving *gld-1*(*op236*)*; vig-1*(*ok2536*) larvae develop an abnormal morphology, as indicated by arrowheads (*p < 0.01, Fisher's exact test). Left panel DIC image, right panel fluorescent image of the same animal that expresses *col-19::GFP*. As a control, wild-type animals are shown at the lower panel.

We performed a genetic screen to search for genes whose inactivation enhances the *gld-1*(*op236*) defect. *gld-1*(*op236*) animals have wild-type germlines at 20°C but are sterile at 25°C [13]. In a whole-genome RNAi feeding screen, we looked for genes whose depletion by RNAi causes sterility at 20°C specifically in *gld-1*(*op236*) mutants but not in wild-type animals (figure 1*a*). Out of over a hundred initial candidates (data not shown), 20 reproducibly showed reduced progeny in the *gld-1*(*op236*) background (figure 1*b*). Of these, deletions were available for seven candidates, and double mutants of five of these with *gld-1* confirmed the reduced progeny or sterility phenotype observed in the RNAi screen. A detailed analysis of synthetic phenotypes with *hecd-1*, *eel-1* and *larp-1* will be described elsewhere. Interestingly, *vig-1* and *nhl-2* were previously identified as modulators of the miRNA pathway [4,5].

We observed that a significant portion of *gld-1*(*op236*)*; nhl-2*(*ok818*) animals are sterile and have stacked oocytes in their germlines and no fertilized embryos in the uterus (electronic supplementary material, figure S1*a*,*b*). Analysis of *gld-1*(*op236*)*; vig-1*(*ok2536*) animals revealed that the entire germline fills with undifferentiated cells and animals become sterile within 12 h of reaching adult stage (electronic supplementary material, figure S1c,d). In addition, embryos laid from these animals show a highly penetrant embryonic lethality phenotype (electronic supplementary material, figure S1e). The sterility phenotypes of the *gld-1*(*op236*)*; nhl-2*(*ok818*) and *gld-1*(*op236*)*; vig-1*(*ok2536*) double mutants confirm the results of our RNAi screen.

Unexpectedly, 29% of the surviving *gld-1*(*op236*)*; vig-1*(*ok2536*) larvae have arrested development and abnormal somatic morphology (figure 1*c,d*). ‘Extruding bumps’ are readily observed at various positions along the body axis of these animals. Using the *col-19::GFP* hypodermal cell marker, we found that hypodermal cells accumulate in these protrusions rather than being regularly spaced along the body axis (figure 1*c*). In addition, double-mutant animals appear shorter and sick. Sterility and embryonic lethality phenotypes have already been described for *dcr-1* (dicer endonuclease) mutants as well as for *alg-1/alg-2* Argonaute double mutants [38–40]. Given this similarity, and as both VIG-1 and NHL-2 modulate miRNA function, we decided to investigate possible genetic interactions between *gld-1* and miRNAs.

### 4.2. *gld-1* genetically interacts with mir-35 family miRNAs

To investigate an interaction between *gld-1* and miRNAs, we started by making double mutants of *gld-1*(*op236*) and *mir-35* family miRNAs. The *mir-35* family of miRNAs comprises eight members (*miR-35*–*42*), which are highly enriched in oocytes [20] and are required for embryonic development. While *mir-35* family mutants do not individually exhibit an observable phenotype, combined mutation of either all or most *mir-35* family members causes severe embryonic and larval lethal phenotypes [20], similar to those we observed in the *gld-1*(*op236*)*; vig-1*(*ok2536*) double mutant. The expression pattern and phenotypes of *mir-35* family miRNAs make them suitable to investigate possible genetic interactions with *gld-1*.

**Figure 2.**
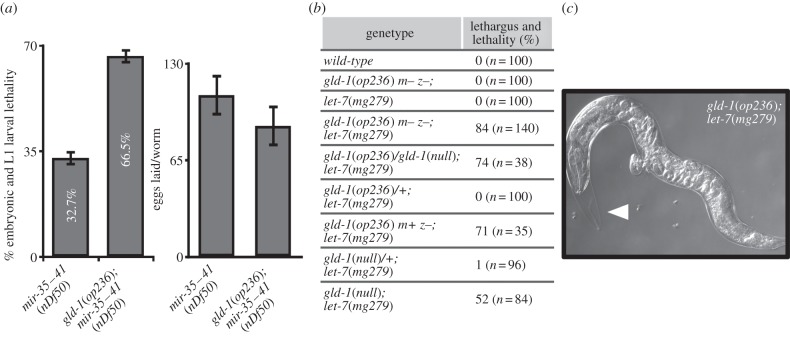
*gld-1* genetically interacts with *mir-35* and *let-7* family miRNAs. (*a*) Adult animals 24 h past L4 stage were allowed to lay eggs and quantitation of embryonic and larval lethality is depicted for *mir-35–41*(*nDf50*) and *gld-1*(*op236*)*; mir-35–41*(*nDf50*) mutants at 20°C (left graph). *gld-1*(*op236*) animals are 100% viable. Number of eggs laid per worm is shown in the right graph. Each experiment was carried out in quadruplicate (*n* > 250), and the percentage of dead eggs and L1 worms was calculated (error bars = s.e.m.). (*b*) Lethality owing to adult-stage lethargus. Number of assayed worms is mentioned in parenthesis. m, maternal genotype; z, zygotic genotype. Synchronized L1 stage animals were grown to adult stage and assayed for lethality owing to internal hatching of embryos. Owing to the sterility of *gld-1*(*null*) animals, *gld-1*(*null*)*; let-7*(*mg279*) phenotype is determined by slow movement and lack of pharyngeal activity during L4 to young adult transition. (*c*) Representative picture of a *gld-1*(*op236*)*; let-7*(*mg279*) worm. The accumulation of late stage embryos is evident. Arrowhead indicates cuticle that failed to shed.

A deletion mutant, *nDf50*, which removes all *mir-35* family miRNAs except for *mir-42*, causes a temperature-sensitive embryonic and early larval lethality [20]. At 20°C, we observed that 33% of *mir-35–41*(*nDf50*) animals die either during embryogenesis or at the L1 larval stage. In *gld-1*(*op236*)*; mir-35–41*(*nDf50*) double-mutants embryonic and larval lethality increases to 67%, indicating a strong genetic interaction between *gld-1* and the *mir-35* miRNA family (figure 2*a*, left). Given that both *mir-35–41*(*nDf50*) and *gld-1*(*op236*)*; mir-35–41*(*nDf50*) mutants lay similar numbers of eggs (figure 2*a*, right), the synthetic interaction between *gld-1* and *mir-35* family miRNAs must specifically affect early embryonic development.

### 4.3. *gld-1* genetically interacts with *let-7* family miRNAs

Next, we decided to investigate whether *gld-1* can genetically interact with other miRNA families. The *let-7* family (*let-7*, *mir-48*, *mir-84*, *mir-241* and *mir-795*) miRNAs are much more studied compared with *mir-35* family miRNAs during *C. elegans* development. One of the phenotypes in *let-7* mutants relates to moulting [41]. *Caenorhabditis elegans* has four larval stages, and each larval stage ends with a moult. Moulting starts with a sleep-like stage called lethargus during which worms slow down feeding and movement. During lethargus, a new cuticle is synthesized, and moulting ends with the removal of the old cuticle [42]. A supernumerary fifth moult has been described in *let-7*(*mg279*)*; mir-84*(*tm1304*) double mutants [41], during which adult animals cease to move and stop pharyngeal activity. Subsequently, affected animals fail to lay eggs and die owing to the internal hatching of embryos.

We did not observe such a phenotype in *gld-1*(*op236*) and in the hypomorphic *let-7*(*mg279*) single mutant, but to our surprise, this phenotype occurred in 84% of *gld-1*(*op236*)*; let-7*(*mg279*) double mutants (figure 2*b* and electronic supplementary material, movie S1). The *mg279* allele has a promoter mutation that reduces *let-7* expression [43]. As previously observed for *let-7*(*mg279*)*; mir-84*(*tm1304*) double mutants [41], *gld-1*(*op236*)*; let-7*(*mg279*) animals with only partially shed cuticles can be observed (figure 2*c*). *gld-1*(*q485*) *null/gld-1*(*op236*)*; let-7*(*mg279*) *and gld-1*(*q485*) *null; let-7*(*mg279*) double-mutant worms show supernumerary moulting phenotypes confirming that the synthetic phenotypes are really caused by mutations of the *gld-1* gene. Heterozygous *gld-1*(*op236*)*/+; let-7*(*mg279*) animals have wild-type appearance (figure 2*b*) consistent with *gld-1*(*op236*) behaving as a recessive allele. *gld-1*(*op236*) *m+ z-; let-7*(*mg279*) (m, maternal genotype; z, zygotic genotype) animals have a comparable phenotype with *gld-1*(*op236*) *m- z-; let-7*(*mg279*) animals (figure 2*b*), showing that maternal contribution of *gld-1* does not affect the supernumerary moulting phenotype. To extend our analysis, we investigated possible genetic interactions of *gld-1*(*op236*) with the remaining *let-7* family members *mir-48, mir-84* and *mir-241*. Forty-two per cent (*n* = 43) of *mir-48 mir-241; mir-84* triple mutants die owing to a burst vulva during the L4 to adult transition reminiscent to the *let-7*(*null*) phenotype [44]. The penetrance of this phenotype is enhanced by *gld-1*(*op236*), with 64% (*n* = 81, *p* = 0.013 Fisher's exact test) lethality observed in the *gld-1*(*op236*); *mir-48 mir-241; mir-84* quadruple mutant. *gld-1*(*op236*) did not affect levels of mature *let-7* miRNA, thereby ruling out the possibility that GLD-1 has an essential, non-redundant role in miRNA processing (see electronic supplementary material, figure S2). Our results show that *gld-1* can genetically interact with the *let-7* miRNA family during somatic development when the *let-7* miRNA pathway is sensitized through mutations of the *let-7* family miRNAs.

### 4.4. *gld-1*(*op236*) affects *let-7* regulation of hypodermal development

**Figure 3.**
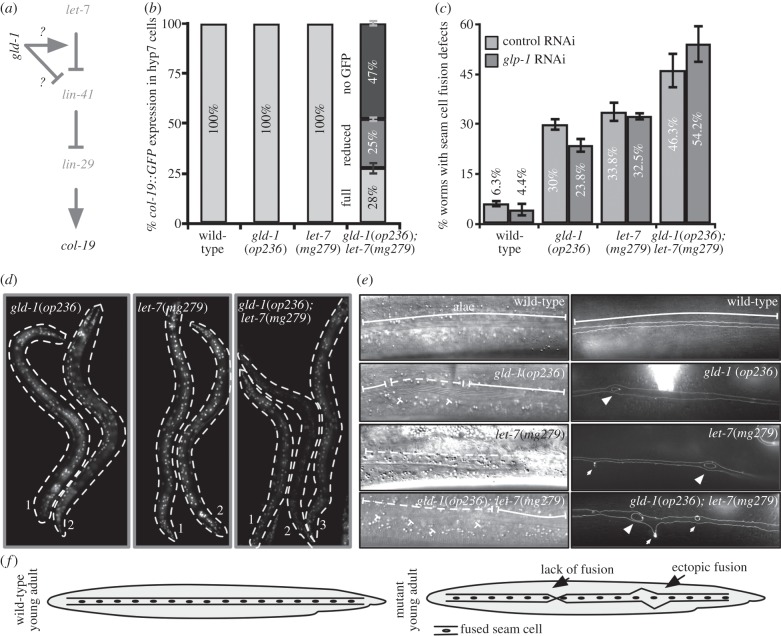
*gld-1* affects the *let-7* regulated hypodermal development (*a*) Simplified diagram of the *let-7* pathway leading to *col-19* expression. (*b*) *col-19::GFP* expression in hypodermal hyp7 cells (error bars = s.e.m. of triplicate results). (*c*) Seam cell fusion defects assayed by the *ajm-1::GFP* junction marker upon either control RNAi or *glp-1* RNAi (error bars = s.e.m. of quadruplicate results, *n* = 20 for each replicate). In *glp-1* RNAi, only the animals without a germline were assayed. (*d*) Representative pictures of *col-19::GFP* expressing worms. Numbers indicate worms. Note the complete absence of signal in worm number 2 in the right panel. (*e*) Representative images of animals showing adult-stage alae and seam cell fusions. In wild-type worms, complete alae and complete seam cell fusion can be seen. Strong ectopic junctions (arrow heads), weak ectopic junctions (small, thin arrows) and lack of junctions (not shown) are observed in *gld-1*(*op236*)*, let-7*(*mg279*) and *gld-1*(*op236*)*; let-7*(*mg279*) worms (right hand panel). In the left-hand panels, partial alae or lack of alae are indicated by dashed lines and ectopic alae are indicated by small T-bars. (*f*) Schematic drawing of the seam cell fusion defects observable by AJM-1::GFP.

In order to better understand the extent of genetic interactions between *gld-1* and the *let-7* miRNA, we focused on the role of *let-7* miRNA in hypodermal development. During the L4 to adult transition, *let-*7 downregulates *lin-41,* a TRIM-NHL domain protein that keeps the transcription factor LIN-29 in an inactive state possibly through mRNA regulation as described for mammalian systems [45]*.* LIN-29 transcriptionally activates adult-stage-specific genes such as collagen *col-19* [46]*.* Either lack of *let-7* or disrupted *let-7* function, causes loss of *col-19* expression owing to increased LIN-41 expression that leads to reduced LIN-29 activity (figure 3*a*) [41]. A transcriptional reporter expressing GFP under the control of the *col-19* promoter reveals that both *gld-1*(*op236*) and *let-7*(*mg279*) single mutants have unaltered *col-19::GFP* expression (figure 3*b*). Interestingly, only 28% of *gld-1*(*op236*)*; let-7*(*mg279*) double-mutant animals have wild-type levels of transgene expression (figure 3*b*) and 47% of double-mutant animals do not express *col-19::GFP* in the hypodermal hyp7 cells (figure 3*d*). *col-19::GFP* expression is not affected in *gld-1*(*op236*)*/+; let-7*(*mg279*) (1.5%; *n* = 66) again indicating recessiveness of *gld-1*(*op236*)*.* Conversely, reduced expression (60.5%; *n* = 114) in *gld-1*(*op236*)*/gld-1*(*q485*)*; let-7*(*mg279*) *strains* indicates that the phenotype is due to a mutation of *gld-1*.

At the end of the L4 larval moult, lateral seam cells fuse and form extracellular structures called alae [47]. The timing of seam cell fusion and alae formation is controlled by *let-7* family miRNAs [43]. Similarly, mutations in *C. elegans* genes encoding AGO (*alg-1, alg-2*) and GW182 proteins (*ain-1, ain-2*) also have seam cell fusion and alae formation defects [18,38]. We assayed seam cell fusion using the AJM-1::GFP junction marker as indicated (figure 3*f*). Analysis of differential interference contrast (DIC) images and the AJM-1::GFP junction marker indicate defects in alae formation and seam cell fusions in *gld-1*(*op236*)*, let-7*(*mg279*) and *gld-1*(*op236*)*; let-7*(*mg279*) animals (figure 3*e*). We next quantified the extent of seam cell fusion defects and found that the incidence of seam cell fusion defects is higher in *gld-1*(*op236*)*; let-7*(*mg279*) double-mutant animals than in single mutants (figure 3*c*). In summary, our combined data suggest that *gld-1* affects hypodermal development in *let-7* mutant background, either by acting through *let-7* or through a parallel pathway.

### 4.5. GLD-1 affects *let-60* signalling

**Figure 4.**
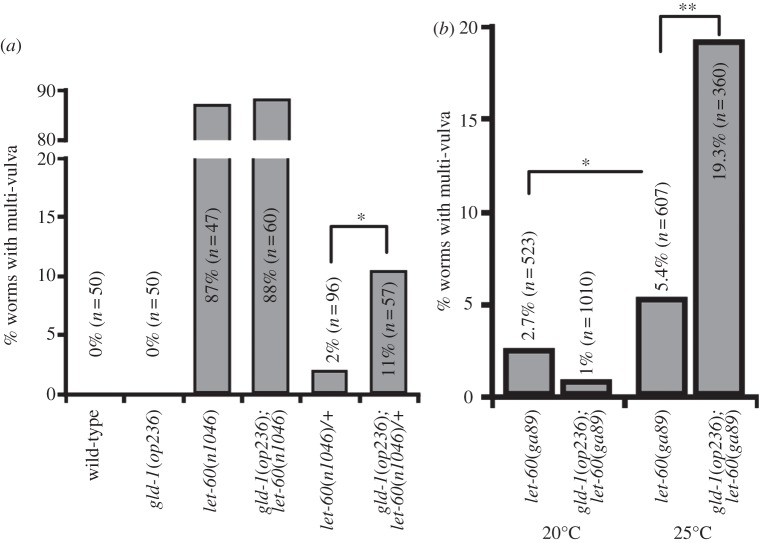
*gld-1*(*op236*) induces vulva formation. (*a*) *gld-1*(*op236*) enhances the multi-vulva phenotype in the heterozygous *let-60*(*n1046*)*/+* gain-of-function background. (*b*) *gld-1*(*op236*)*; let-60*(*ga89*) shows increase in multi-vulva formation when switched from 20 to 25°C (*n* > 40 for each genotype, *p < 0.05, **p < 0.01 by Fisher's exact test).

To check whether the genetic interactions of *gld-1* with the *let-7* miRNA family are restricted to the hypodermal development, we looked into the let-60/RAS pathway that functions during vulva formation [48]. *mir-84* and *let-7* antagonize let-60/RAS signalling in vulval precursor cells that are not destined to form the vulva. Such regulation can be assessed *in vivo* using let-60/RAS gain-of-function alleles that induce ectopic vulva formation by triggering excessive MAP kinase signalling. The system is sensitized by maintaining the *let-60*(*n1046*) gain-of-function allele in a heterozygous state or switching *let-60*(*ga89*) temperature-sensitive gain-of-function allele from 20 to 25°C. *gld-1*(*op236*)*; let-60*(*n1046*)*/+* (figure 4*a*) and *gld-1*(*op236*)*; let-60*(*ga89*) (figure 4*b*) double mutants have an increased incidence of forming multiple vulvae, indicating that GLD-1 also affects vulva induction.

### 4.6. Germline expression of *gld-1* is not necessary for somatic phenotypes

GLD-1 protein expression has previously been reported in the germline [7]. We therefore wished to determine whether *gld-1* expression also occurs in somatic tissues, and whether the genetic interactions we observe during somatic development depend on a germline. To determine whether a germline is required for seam cell fusion defects in *gld-1*(*op236*) and *gld-1*(*op236*)*; let-7*(*mg279*) animals, we used RNAi to inactivate *glp-1*, which is essential for germline development [49]. We observed that the defective seam cell fusion phenotype occurs to a similar extent in animals lacking a germline as in those with a germline (figure 3*c*). Furthermore, somatic expression of an mCherry::H2B transcriptional reporter (the histone fusion used to focus diffuse, low level cytoplasmic GFP expression to the nucleus) under the control of the *gld-1* promoter and the *gld-1 3′UTR* was mainly localized to the head, tail and ventral side of the animals (see electronic supplementary material, figure S3). DIC microscopy analysis indicates that most of the positive cells are neuronal cells in the head and tail ganglia and the ventral nerve cord. Interestingly, *let-7* and at least one *let-7* target, *hbl-1*, is also reported to have a similar expression pattern [50], although it is not known to what extent *let-7* miRNA phenotypes require hypodermal or neuronal expression. It is likely that high levels of background fluorescence masked low levels of *gld-1* reporter expression in other cell types. We could not confirm this localization pattern by antibody staining owing to background problems. However, we generated transgenic animals expressing full-length *gld-1* fused to histone::GFP by an operon linker, generated by single copy insertion using the MosSCI technique [29,51,52]. This transgene shows the same pattern of somatic expression, shows expression in embryos and rescues *gld-1*(*null*) in the germline (electronic supplementary material, figure S4). Even though these reporter constructs might not exactly represent endogenous GLD-1 expression, together with our genetic results, they suggest somatic roles for *gld-1.*

### 4.7. Overexpressing a *lin-41* 3′UTR construct acts as a ‘sponge’ to sequester *let-7* miRNA and provides a sensitized system to assay GLD-1 activity

**Figure 5.**
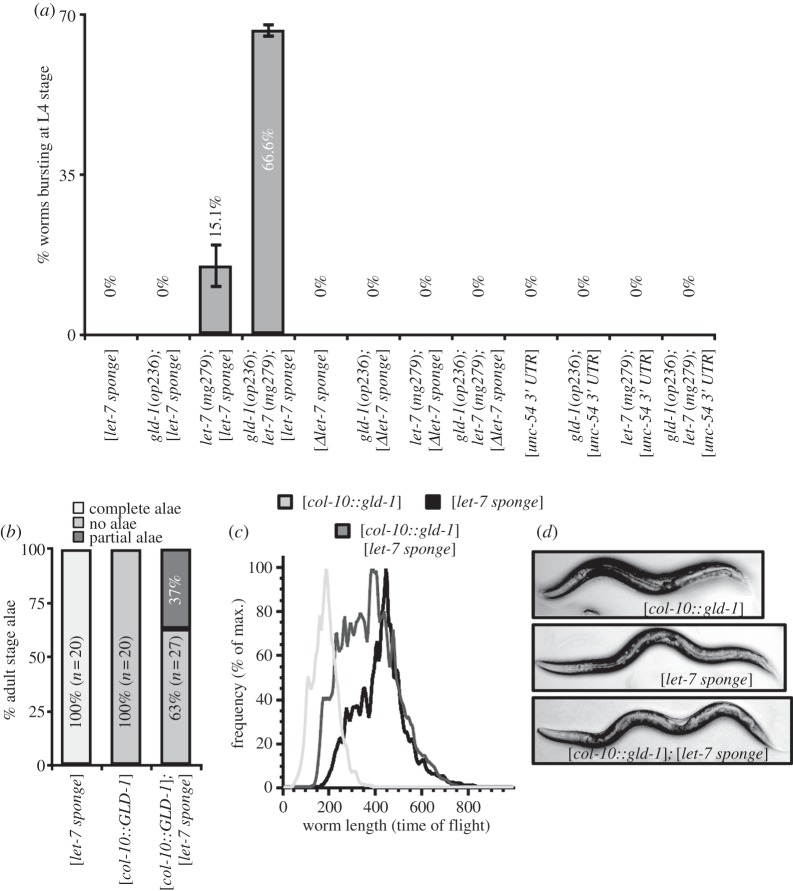
A *let-7 sponge* transgene generates a sensitive system to test miRNA function. (*a*) *let-7* sponge (*col-10::GFP::lin-41 3′UTR*) causes mild bursting phenotype in *let-7*(*mg279*) mutants. Bursting dramatically increases in *gld-1*(*op236*)*; let-7*(*mg279*); *[let-7 sponge*] animals (error bars = s.e.m.). Using *lin-41 3′UTR* with deleted *let-7* binding sites ([**Δ*let-7sponge*]) or [*unc-54 3′UTR*] in the sponge construct doesn't cause any phenotypes. (*b*) *gld-1* expression under the control of the *col-10* promoter causes lack of adult-stage alae. *let-7* sponge partially rescues the alae defects in *col-10::GLD-1* expressing animals. (*c*) *col-10::GLD-1* expressing animals have a dumpy phenotype and short size. *let-7* sponge partially rescues the dumpy phenotype and the short size of the animals are rescued to wild-type levels. The relative length of the animals is measured through time of flight by a COPAS biosorter (*n* > 2000). (*d*) Representative DIC images of animals expressing *col-10::GLD-1*, *let-7 sponge* and *col-10::GLD-1; let-7 sponge*.

One of the targets of *let-7* miRNA during larval development is the *lin-41* mRNA [45]. Hypodermal defects in *gld-1*(*op236*)*; let-7*(*mg279*) could be the result of mis-regulation of *lin-41* mRNA. To investigate this possibility, we generated a single copy MosSCI insertion of *GFP::lin-41–3*′*UTR* fusion construct under the control of the *col-10* promoter that ensures expression in hypodermal tissues. We did not observe any phenotype (figure 5*a*) in wild-type or in *gld-1*(*op236*) animals. However, *let-7*(*mg279*) mutants showed a low penetrance bursting through the vulva phenotype reminiscent to *let-7*(*null*) phenotype (figure 5*a*). We likened this observation to a sponge-like effect of the *GFP::lin-41–3*′*UTR* towards *let-7* miRNA. miRNA sponges are complementary target sequences that can sequester the miRNA from its endogenous target [53]. In *C. elegans*, such a sponge was used for the *lin-4* miRNA [54]. The penetrance of the vulva-bursting phenotype is dramatically enhanced in *gld-1*(*op236*)*; let-7*(*mg279*) double mutants expressing the *GFP::lin-41–3*′*UTR* (*let-7 sponge*) (figure 5*a*). Thus, *gld-1*(*op236*) specifically enhances the *let-7*-dependent phenotypes, and the extent of genetic interactions between *gld-1* and the *let-7* miRNA pathway becomes more evident when the *let-7* miRNA pathway is further compromised. Expression of a *let-7 sponge* with a deletion of the 3 *let-7* binding sites or expression of the unrelated *unc-54* 3′UTR did not cause any bursting phenotype in *let-7*(*mg279*) and *in gld-1*(*op236*)*; let-7*(*mg279*) animals supporting the specificity of the *let-7 sponge* and the interactions between *gld-1* and *let-7* miRNA (figure 5*a*).

We next expressed *gld-1* under the control of the *col-10* promoter in the hypodermis to investigate whether such expression of *gld-1* might cause any phenotype associated with the loss of *let-7* targets. *col-10::GLD-1* expression in the hypodermis caused a loss of adult-stage alae (figure 5*b*) and a dumpy phenotype (shortened size; figure 5*c,d*). A dumpy phenotype also occurs following mutation of *let-7* targets such as *lin-41* [45]. Co-expression of the *let-7* sponge partially rescues the dumpy and loss of alae phenotypes (figure 5*b–d*). Based on these results, we cannot exclude the possibility that *gld-1* and *let-7* miRNA function in parallel pathways during the hypodermal development. Equally, GLD-1 expression in the hypodermis might cause unrelated phenotypes. However, another likely interpretation of these experiments is that GLD-1 and *let-7* act in conjunction to excessively repress target mRNAs possibly in the same pathway, and that reducing the ‘dose’ of *let-7* using the sponge alleviates target gene repression.

### 4.8. SILAC in nematodes identifies proteome wide changes in *gld-1* and *let-7* mutants

**Figure 6.**
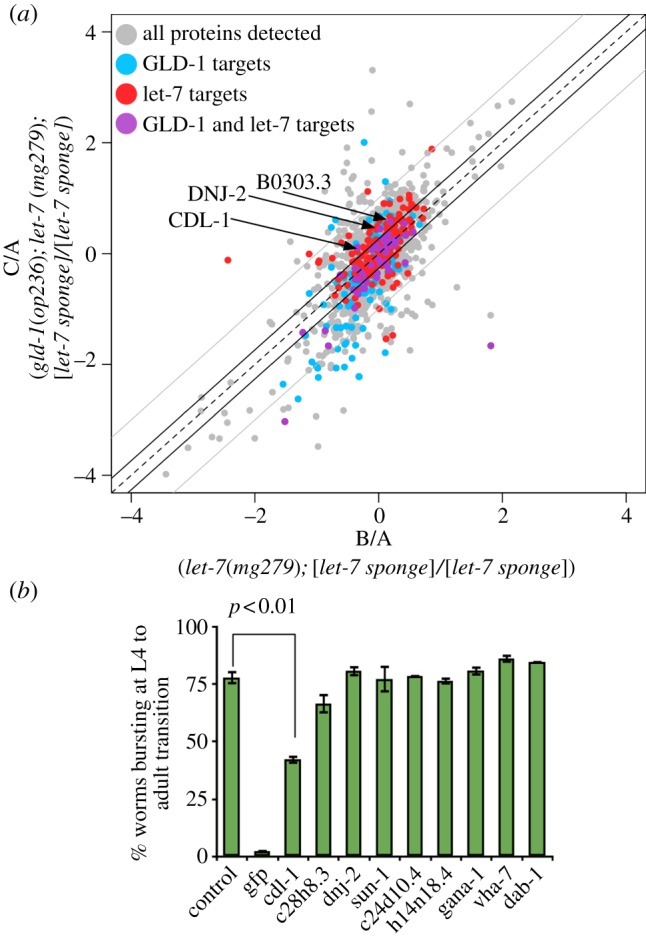
SILAC-based proteomics in *let-7* and *gld-1* mutants. (*a*) log_2_ relative abundances of 2179 proteins in *let-7*(*mg279*); [*let-7 sponge*] (*x*-axis) and *gld-1*(*op236*)*; let-7*(*mg279*); [*let-7 sponge*] (*y*-axis) animals compared with [*let-7 sponge*] animals alone. Solid black and grey lines indicate 1.2-fold and twofold thresholds, respectively. Dots represent 2179 proteins. Among them GLD-1 targets [55,56] are coloured blue, mirWIP database *let-7* target predictions [57] are coloured red, and the possible GLD-1 and *let-7* co-targets based on these lists are coloured purple. The remaining proteins are coloured in grey. CDL-1, DNJ-2 and B0303.3 are possible GLD-1 and *let-7* targets that are upregulated more than 1.2-fold (arrows). (*b*) RNAi-mediated knockdown of 9 genes upregulated in the *gld-1*(*op236*)*; let-7*(*mg279*); [*let-7 sponge*] animals (RNAi is done in the same strain). We picked six genes upregulated more than twofold and are GLD-1 or predicted *let-7* targets (red and blue spots above the twofold line) and three genes upregulated more than 1.2-fold that are GLD-1 and predicted let-7 targets (purple spots above the 1.2-fold line). Empty vector RNAi and GFP RNAi are used as negative and positive controls respectively. B0303.3 RNAi results are omitted due to the early larval lethality in these animals (error bars = s.e.m. of triplicate, *p*-values calculated using Fisher's exact test).

Our results suggest that GLD-1 and *let-7* synergistically affect animal development. We wanted to determine whether the phenotypes we observed are due to the mis-regulation of either a single or small number of genes, as opposed to affecting multiple target genes. In addition, we wanted to determine whether GLD-1 and the let-7 miRNA regulate distinct or same targets. To address this question, we used nematode SILAC to systematically quantify protein levels [34,36]. The strongest genetic interaction between *gld-1* and *let-7* occurs in the *let-7 sponge* system (figure 5*a*). Thus, to assess changes in the proteome of the respective strains, we used the following experimental set-up. For the SILAC experiment synchronized L1 larvae of three strains, namely (A) [*let-7 sponge*], (B) *let-7(mg279*); [*let-7 sponge*] and (C) *gld-1*(*op236*)*; let-7*(*mg279*)*;* [*let-7 sponge*] were grown up to the young-adult stage until the bursting phenotype just becomes visible and subjected to quantitative mass spectrometry (figure 6*a*). As the animals expressing *let-7 sponge* alone do not display any phenotype, we considered them as the baseline similar to using wild-type. Thus, by comparing the animals with a weak phenotype (B) to animals with a strong phenotype (C), we aimed to identify proteins whose expression change might be responsible for the bursting through the vulva phenotype and help explain the interaction between *gld-1* and the *let-7* miRNA. In a SILAC experiment, the relative ratios of two sets of proteins can be measured by differential isotope labelling. We then determined proteins differentially expressed when B was compared with A. At the same time, we also determined differentially expressed proteins comparing C with A. As a control, we analysed the levels of GFP expression under the control of *lin-41* 3′UTR sponge by Western blotting. GFP levels are higher in the *let-7*(*mg279*)*; let-7* sponge animals, compared with the *let-7 sponge* only (B to A; electronic supplementary material, figure S5*a*). We identified a similar rise in GFP levels in SILAC experiments, and the level of GFP was further increased in *gld-1*(*op236*)*; let-7*(*mg279*)*; let-7 sponge* animals (C to A; electronic supplementary material, figure S5*b*). This result confirms the sensitivity of our SILAC-based approach. In the B/A comparison, 2708 proteins passed our specificity criteria, whereas 2927 passed in the C/A comparison (see Materials and methods). We focused on comparing the abundance of the 2179 proteins that were reliably detected in both datasets. The ratios of (C) *gld-1*(*op236*)*; let-7*(*mg279*)*;* [*let-7 sponge*] protein to (A) [*let-7 sponge*] protein shown on the *y*-axis are compared with the (B) *let-7* (*mg279*)*;* [*let-7 sponge*], with (A) [*let-7 sponge*] ratios depicted on the *x*-axis (figure 6*a*).

Among these 2179 proteins, 252 overlap with the 1084 previously described GLD-1 targets (figure 6*a*, coloured in blue) [55,56]. Proteins (239) overlap with 1322 predicted *let-7* targets (figure 6*a*, coloured in red, mirWIP database [57]). Fifty-four proteins are predicted to be both GLD-1 and *let-7* targets (figure 6*a*, coloured in purple). The relative abundance of the majority of suspected GLD-1 and *let-7* co-targets do not change when the C/A ratios are compared with the B/A ratios. However, three such candidate proteins, namely *cdl-1*, *dnj-2* and B0303.3 were more prominently upregulated in C/A.

We tested whether the depletion of these three proteins suppresses the vulva-bursting phenotype of the *gld-1*(*op236*)*; let-7*(*mg279*)*;* [*let-7 sponge*] strain (figure 6*b*). Targeting the [*let-7 sponge*] which is a *col-10::GFP::lin41 3′UTR* construct by GFP RNAi lead to a complete suppression of the vulva-bursting phenotype and thus served as a positive control. The depletion of one of the candidates, namely *cdl-1* lead to a reduced vulva-bursting phenotype consistent with the notion that the upregulation of CDL-1 in the *gld-1*(*op236*)*; let-7*(*mg279*) background might contribute to the vulva-bursting phenotype. Indeed, *cdl-1 3′UTR* harbours a GLD-1 and a *let-7* binding site (electronic supplementary material, figure S7). CDL-1 is a histone mRNA hairpin binding protein required for expression of histones [58–61]. It is expressed in all somatic cells with strong expression in proliferating cells such as hypodermal cells, intestinal cells and the germ cells [59]. CDL-1 is required for larval development and affects vulva morphology [59,61]. However, the suppression of the vulva-bursting phenotype by CDL-1 RNAi in *gld-1*(*op236*)*; let-7*(*mg279*)*;* [*let-7 sponge*] animals is not complete. This indicates that other factors also contribute to the vulva-bursting phenotype.

### 4.9. Identification of GLD-1-containing complexes

**Figure 7.**
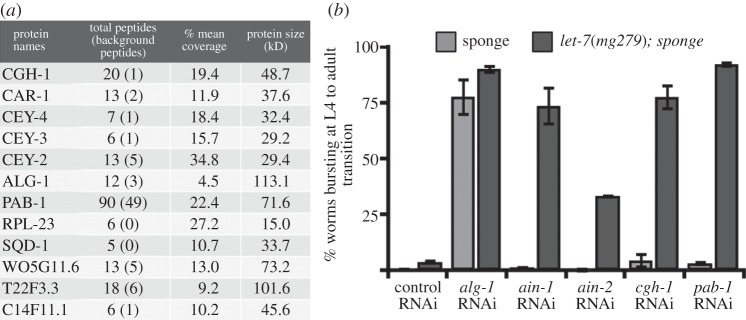
Protein interactors of GLD-1 and their effect on hypodermal development upon RNAi depletion. (*a*) List of protein interactors identified in both anti-GLD-1 antibody IPs in wild-type animals and anti-GFP IPs in *gld-1::GFP* expressing animals. Total peptides detected in Ab IPs and background peptides detected in mock IPs together with % coverage of the peptides are indicated. Original data is in electronic supplementary material, figure S6. (*b*) Quantification of bursting phenotype upon RNAi knockdown of indicated genes in *let-7 sponge* (sponge, grey) and *let-7*(*mg279*)*; let-7 sponge* (green) genetic backgrounds (error bars = s.e.m., *n* > 50 for each replicate).

Our data are consistent with GLD-1 either directly or indirectly interacting with the miRNA pathway. To investigate this genetic interaction at biochemical level, we next aimed to purify proteins that interact with GLD-1. We used GLD-1 antibodies to immunoprecipitate GLD-1 complexes in wild-type animals and used mass spectrometry to identify GLD-1 interactors. To verify the specificity of GLD-1 interactions, we also pulled down GLD-1 using a GFP-binder from animals expressing a GLD-1::GFP fusion protein and focused our subsequent analysis on proteins specifically pulled down in both purification approaches (figure 7*a*). Among the GLD-1 interactors identified, many are associated with RNA and some are involved in miRNA-mediated gene regulation (figure 7*a*). We identified the CGH-1 helicase, CAR-1 and the Y-box domain proteins CEY-2, CEY-3 and CEY-4, all previously reported to be components of a complex localized to RNP granules in the *C. elegans* germline, and that are likely to function in translational repression [62]. Importantly, CGH-1 is proposed to act in miRNA-mediated gene expression. Depletion of *cgh-1* enhances the defects of *let-7* family mutants and CGH-1 biochemically interacts with ALG-1, AIN-1 and NHL-2 [5]. Indeed, ALG-1 also co-purified with GLD-1 in our experiments (figure 7*a*). Other proteins identified as GLD-1 interactors include PAB-1 and SQD-1. PAB-1 is a *C. elegans* poly(A) binding protein and also a component of AIN-1, AIN-2 and CGH-1 complexes, suggesting a role in translational regulation and miRNA-mediated repression [18,63,64]. SQD-1 is the *C. elegans* orthologue of the *Drosophila* squid hnRNP protein, previously identified as an AIN-2 and *mir-35* miRNA-associated protein [18,65]. PAB-1, CGH-1, CAR-1, CEY-1-4 and SQD-1 were also identified as GLD-1 protein interactors in a recent study [66]. In the same study, interaction of CGH-1 with GLD-1 was shown to depend on the presence of RNA. Considering all the GLD-1 interactors have RNA-binding domains, the presence of RNA might be essential in all these interactions. In summary, GLD-1 interactors identified in this and previous studies associate with proteins known or suspected to be involved in miRNA-mediated gene repression.

We next investigated whether GLD-1 interactors affect *let-*7 miRNA function similar to GLD-1. We therefore assessed the extent of vulva-bursting upon RNAi of those interactors in *let-7 sponge* and *let-7*(*mg279*)*; let-7 sponge* animals. RNAi against *alg-1, ain-1* and *ain-2*, core miRNA components, were used as positive controls. As expected, *alg-1* RNAi induces a strong vulva-bursting phenotype in both *let-7 sponge* and *let-7*(*mg279*)*; let-7 sponge* animals (figure 7*b*). *ain-1* and *ain-2* RNAi induced the vulva-bursting phenotype only in the sensitive *let-7*(*mg279*)*; let-7 sponge* animals. Among the GLD-1 interactors besides *alg-1* RNAi, *cgh-1* and *pab-1* RNAi also induced a strong vulva-bursting phenotype in the *let-7*(*mg279*)*; let-7* sponge animals, supporting their role in miRNA function.

## 5. Discussion

The starting point of our work was the identification of two miRNA effector genes, *vig-1* and *nhl-2* as genetic enhancers of *gld-1*(*op236*) (figure 1). Both genes had previously been shown to affect miRNA function [4,5], although not in the germline. It is possible that the germline phenotypes we observed are related to a miRNA-associated function, although it is equally likely that VIG-1 and NHL-2 may, either directly or indirectly, affect GLD-1 function, or else may act in conjunction with GLD-1 to mediate translational repression of GLD-1 targets in the germline. Different phenotypes observed in the *gld-1*(*op236*)*; vig-1*(*ok2536*) and the *gld-1*(*op236*)*; nhl-2*(*ok818*) double mutants suggest that *vig-1* and *nhl-2* function in different pathways in the germline. We will investigate these possibilities in future studies.

Intrigued by the genetic interactions with those miRNA effector genes, we investigated possible roles of *gld-1* in multiple miRNA-mediated pathways and uncovered novel roles for *gld-1* during the somatic development of *C. elegans.* Given that *gld-1* phenotypes and expression were previously only described for the germline, we were surprised to find GLD-1 phenotypes associated with somatic development. Enhancement of *mir-35* family embryonic and larval lethal phenotypes may be explained by perturbation of maternal mRNA pools derived from *gld-1*(*op236*) germlines that may enhance *mir-35* phenotypes. However, it is unlikely that such a model can explain the genetic interactions we observed between *gld-1* and *let-7* family miRNAs. *let-7*-related phenotypes arise much later during development, making a mechanism involving the maternal contribution of miRNAs unlikely. Indeed, we show that *gld-1*(*op236*) *m+ z-* animals have a comparable phenotype with *gld-1*(*op236*) *m- z-* animals (figure 2). Furthermore, we observed that *let-7* phenotypes are enhanced by *gld-1* even when *glp-1* RNAi animals lacking a germline were analysed. Finally, recent data published by the modENCODE consortium indicate that *gld-1* is transcribed in *glp-1* mutants that lack a germline [67]. Consistent with this, using two independently generated reporters we observed *gld-1* reporter expression in somatic tissues. Unfortunately, GLD-1 antibody staining was not successful owing to unspecific background in somatic tissues.

We focused on the *let-7* miRNA pathway, whose components have been much more characterized in *C. elegans*. By using already established tools, we could show that *gld-1* affects multiple *let-7* miRNA regulated pathways (figures 2–4). Importantly, we generated a sensitized system using a *let-7 sponge* and showed that *gld-1*(*op236*) specifically enhances *let-7* loss-of-function phenotypes (figure 5). Our *let-7 sponge* system confirms the notion that the miRNA pathways are highly redundant. *let-7* miRNA levels in wild-type animals are sufficient to regulate both the endogenous targets and also an additional transgene target (*let-7 sponge*). However, when the *let-7* miRNA levels are limiting, such as in the hypomorphic *let-7*(*mg279*) mutants, endogenous targets are not efficiently dealt with when the *let-7* sponge is present (figure 5*a*). Only in this ‘very sensitive’ situation, a role for *gld-1* in the *let-7* miRNA pathway becomes apparent. This finding can be explained by the robustness and redundancy of the *let-7* miRNAs and also by the target genes whose mis-regulation is well tolerated. When we expressed GLD-1 specifically in the hypodermis, we observed defects in alae formation and dumpy animals. Even though these phenotypes might be unspecific, as we do not know whether GLD-1 is expressed in this tissue, co-expressing the *let-7 sponge* partially suppresses these phenotypes. This further supports the involvement of *gld-1* either in the *let-7* pathway or in a parallel pathway. This hypothesis is further supported by the functions of the GLD-1 interactors we identified (figure 7): ALG-1 is a core component of the miRNA machinery [38], and CGH-1 can interact with ALG-1 to modulate miRNA-mediated gene regulation [5]. PAB-1 interacts with AIN-1/2, core miRISC components [18,63], and PAB-1 homologues are required for miRNA-mediated gene regulation in mammalian cells [68]. We have shown that the GLD-1 interactors CGH-1 and PAB-1 affect *let-7* miRNA function (figure 7).

Hundreds of genes are regulated by GLD-1, and it is likely that the expression of an even higher number of genes is modulated by miRNAs. In this context, how can we explain the genetic and biochemical interactions we observe? To address this question, we took a state of the art proteomics approach based on SILAC in nematodes. With this approach, we were able to detect the differential expression of more than 2000 proteins, and we were able to compare the relative abundance of these proteins in animals with weak and strong phenotypes. When we tested some of the upregulated proteins for suppression of the vulva-bursting phenotype associated with the *gld-1*(*op236*)*; let-7* (*mg279*)*;* [*let-7 sponge*] strain, we detected a strong suppression with the RNAi-mediated knockdown of *cdl-1* gene. *cdl-1* is a predicted *let-7* miRNA target and it was identified as a GLD-1 target [55–57]. Thus, *cdl-1* is a strong candidate to be co-regulated by both *let-7* and GLD-1. However, we cannot rule out the possibility that the *cdl-1* upregulation is not directly controlled by *let-7* or GLD-1 and it may arise owing to secondary effects. Either way, we can conclude that CDL-1 upregulation in a *let-7* and GLD-1-dependent manner is in part responsible for the vulva-bursting phenotype.

Overall, we suggest two possible mechanisms for GLD-1 and miRNA interactions that are not mutually exclusive and may act in parallel. First, mutation of both GLD-1 and miRNAs could mis-regulate several targets within a single pathway, leading to the observed phenotypes. For instance, moulting defects in *let-7* mutants are partly due to mis-regulation of the nuclear hormone receptors *nhr-23* and *nhr-25* [41], and *nhr-23* is a predicted GLD-1 target [55,56]. We could not detect NHR-23 in our SILAC experiments. Similarly, some of the phenotypes we observed (figure 5*b*) might be explained by negative regulation of LIN-28 by GLD-1 as it was reported for the germline [55]. However, we did not detect changes in LIN-28 protein levels, which is why we did not follow up on this lead.

A second possibility is that GLD-1 and miRNAs may co-regulate a subset of mRNAs. The interactions we observed between GLD-1, CGH-1, PAB-1 and ALG-1 in GLD-1 complexes support such a mechanism. Consistent with this model, GLD-1 appears to associate with several 3′UTRs that are known or predicted miRNA targets in *C. elegans*. For example, the potential *mir-35* targets, *lin-23* and *gld-1* [69], have also been identified as GLD-1 targets [55]. Similarly, *lin-28* and *ztf-7* are *let-7* miRNA targets [70,71] and these genes have also been identified as GLD-1 targets [55].

We are aware that biochemical interactions reflect total animal extracts and that most GLD-1 protein expression is likely to occur in the germline. Thus, the association of GLD-1 with 3′UTRs in somatic cells may be underrepresented in biochemical experiments aimed to determine GLD-1 targets [55]. GLD-1 has been extensively studied in the germline and consensus-binding motifs have been defined [55,72]. We also know that GLD-1 appears to bind RNA as a dimer and that GLD-1 RNA binding is enhanced when multiple binding sites are available [72]. Still, we know next to nothing regarding how GLD-1 actually confers translational repression. It is intriguing that several proteins previously associated with miRNA regulation biochemically interact with GLD-1. CGH-1 interacts with the miRISC complex and GLD-1 in an RNA-dependent manner [5,66], and therefore raises the possibility of being the mediator between the two translational repression mechanisms. In a recent study, one of the *C. elegans* poly(A) binding proteins, PABP-2, was shown to antagonize *let-7* miRNA function [73]. In our study, we show that PAB-1 is required for proper *let-7* function and this is in line with the interactions between PAB-1 and AIN-1 [63]. These results indicate that different poly(A) binding proteins may affect miRNA function in opposite ways and perhaps these effects depend on other RNA-binding proteins like GLD-1. We therefore speculate that the mechanisms of GLD-1-mediated translational repression and miRNA-mediated translational repression may overlap. It will be interesting to investigate this potentially-shared mechanism in the future.

By taking advantage of multiple genetically sensitized miRNA pathways that act during *C. elegans* development to reliably assess subtle changes in gene expression, we could implicate GLD-1 in miRNA-mediated gene regulation. The mammalian GLD-1 homologue QKI has been reported to co-localize and interact with ALG2 [74], and recently QKI was shown to directly interact with and stabilize miR-20a, revealing a role for QKI in tumour suppression [75]. In a more recent study, two QKI isoforms were shown to regulate miR-7 expression in glial cells [76]. Studying the interactions between GLD-1- and miRNA-mediated gene regulation can thus reveal important regulatory interactions occurring during animal development.

## Supplementary Material

Supplementary File

## Supplementary Material

Table S1
