# Supplementary material for: RNA-binding protein GLD-1/quaking genetically interacts with the mir-35 and the let-7 miRNA pathways in Caenorhabditis elegans
Source: Open Biol. 2013 Nov;3(11):130151. doi: 10.1098/rsob.130151 (PMC3843822; doi:10.1098/rsob.130151)
Supplement: Supplementary File [file rsob130151supp1.pdf]

Figure S1

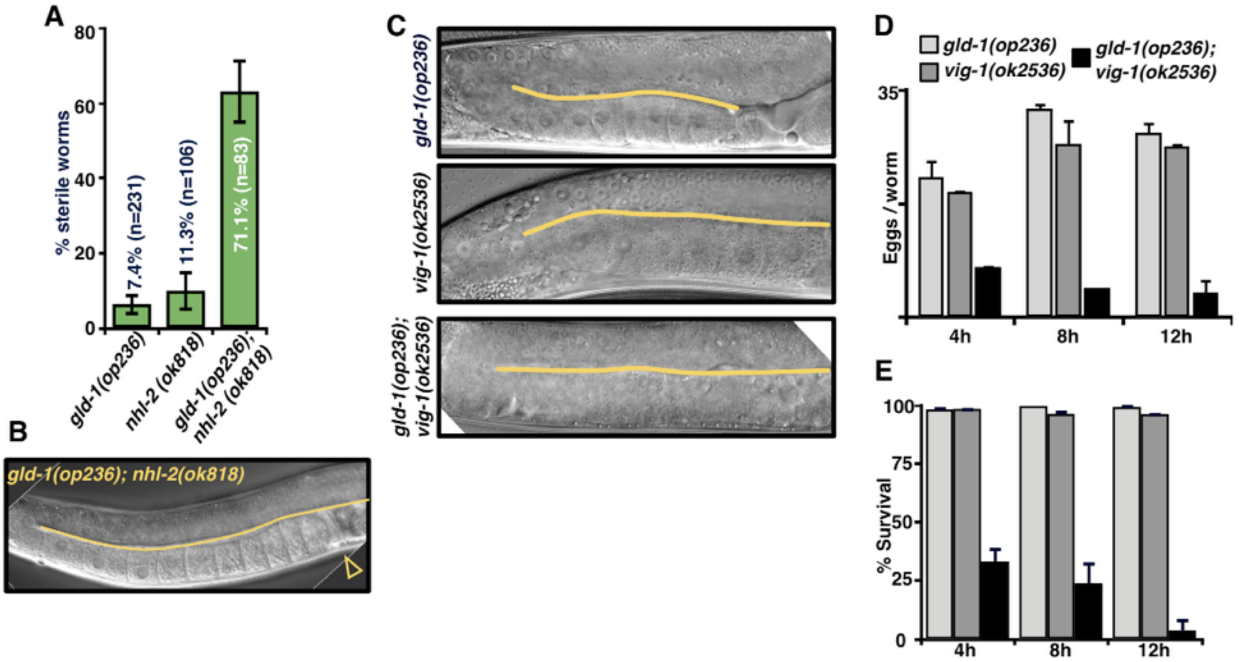

Figure S2

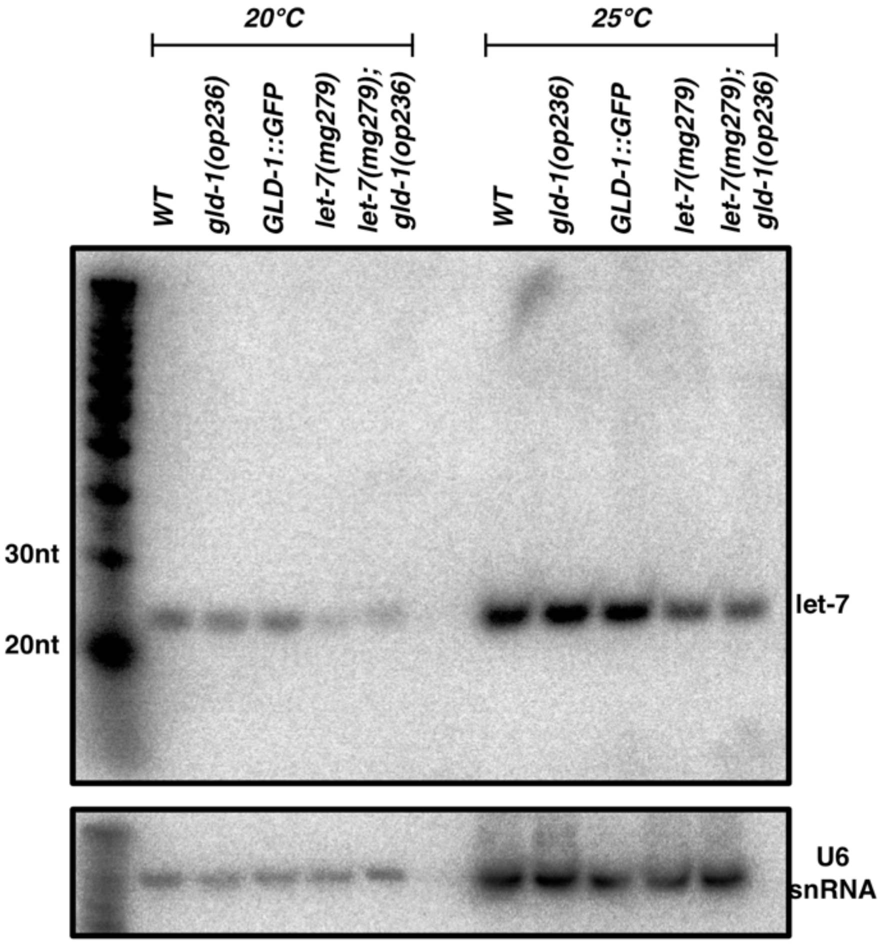

Figure S3

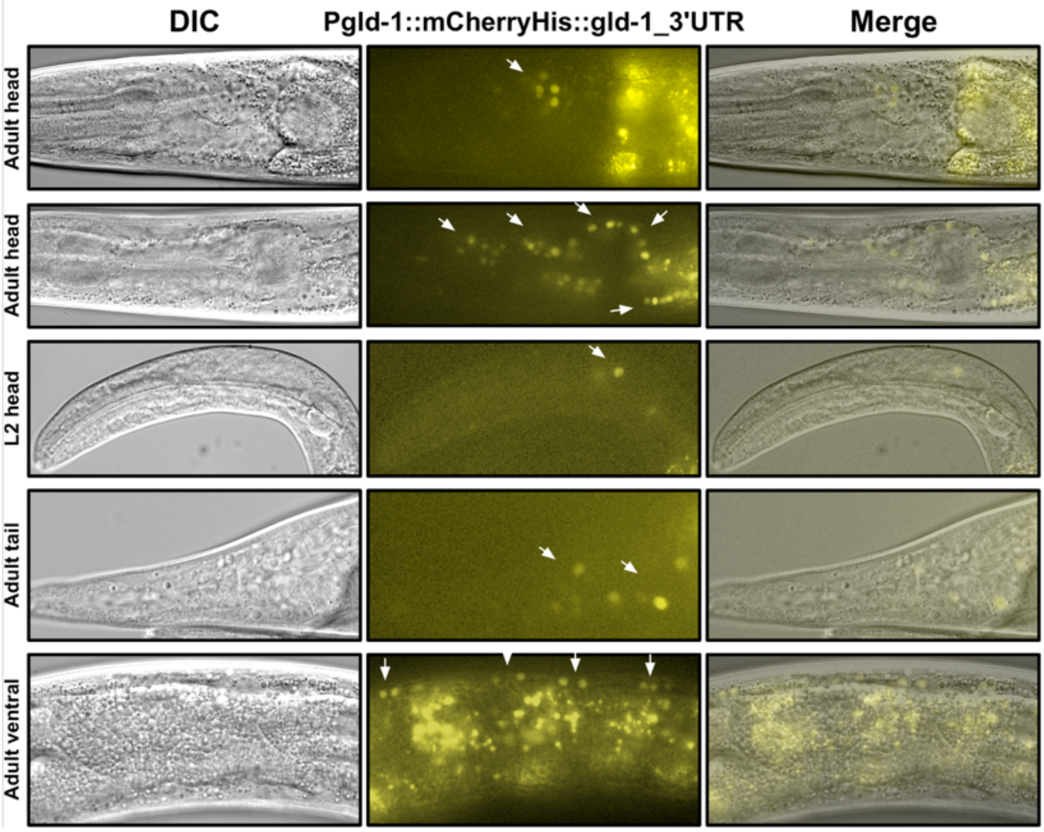

Figure S4

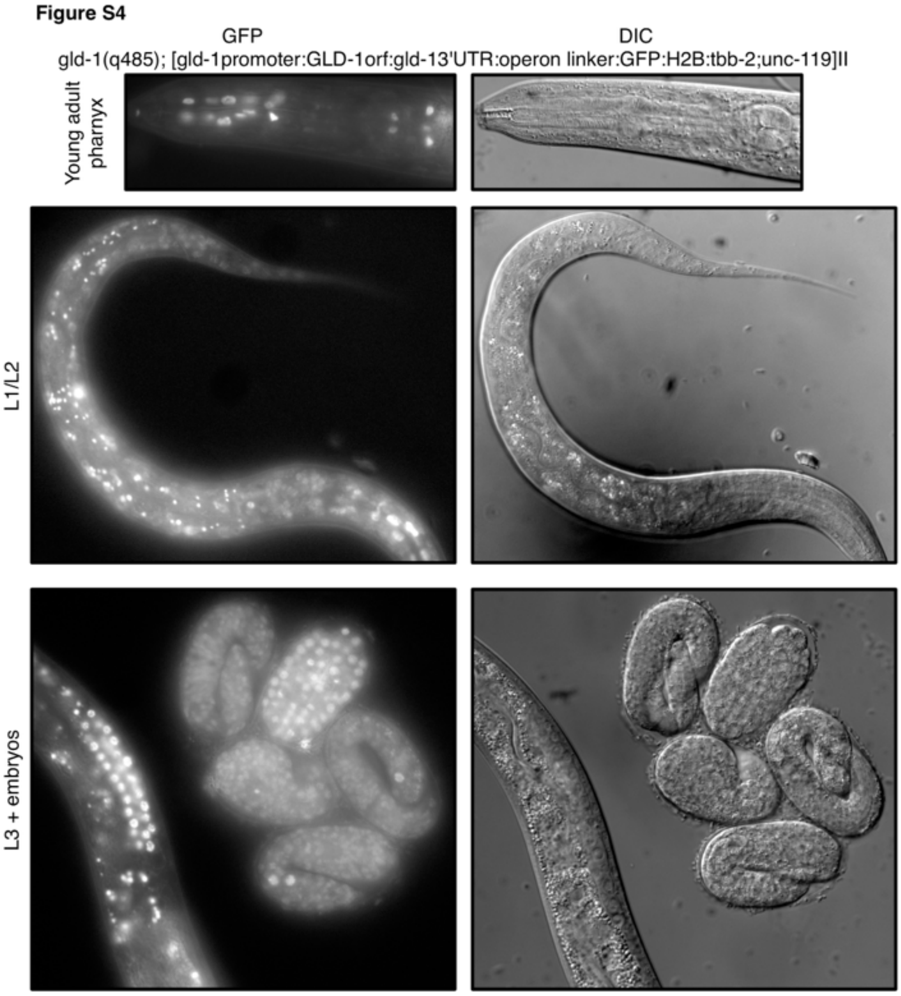

Figure S5

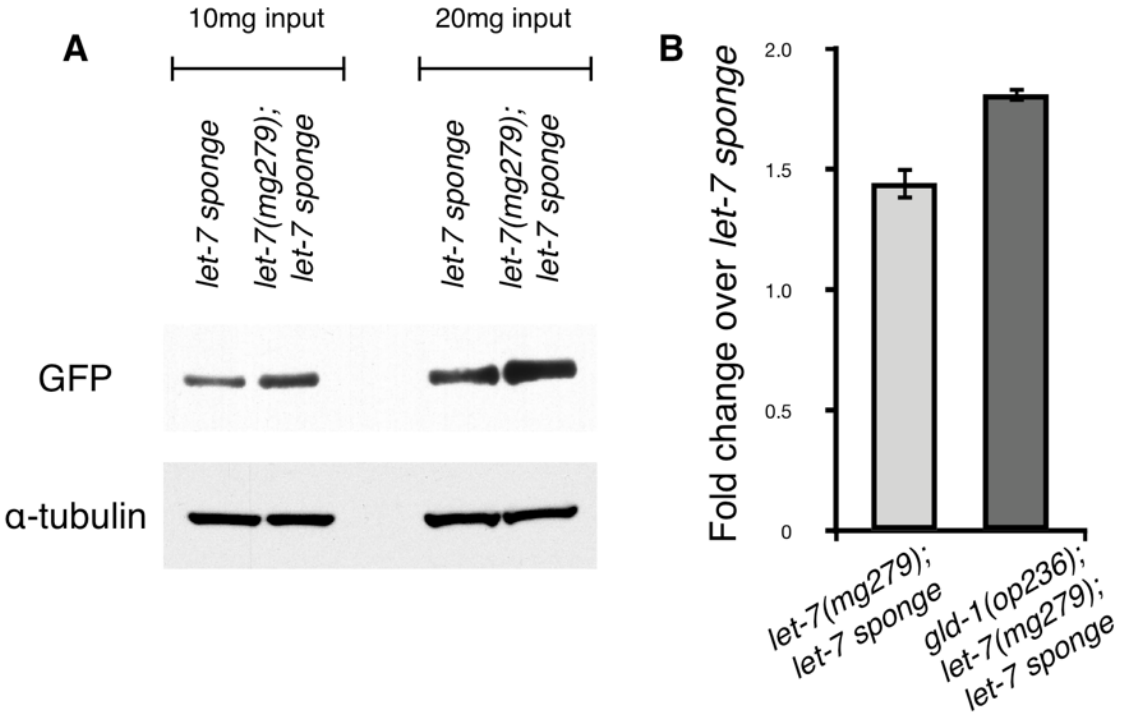

A
10mg input
20mg input
let-7 sponge
let-7(mg279); let-7 sponge
let-7 sponge
let-7(mg279); let-7 sponge
GFP
α-tubulin
B
Fold change over let-7 sponge
2.0
1.5
1.0
0.5
0
let-7(mg279); let-7 sponge
gld-1(op236); let-7(mg279); let-7 sponge

Figure S6

Wormbase Gene IDs	Gene names	GFP-IP1	GFP-IP2	Ab-IP1	Ab-IP2	GFP-IP1-peptides		GFP-IP2 peptides		Ab-IP1 peptides		Ab-IP2 peptides		Total peptides in IP	Total background peptides	% Mean Coverage	Protein size(kD)
						gld-1	bc	gld-1	bc	gld-1	bc	gld-1	bc				
WBGene00001595	gld-1	+	+	+	+	43		28		5		71	10	147	10	24.75	53.711
WBGene00003902	pab-1	+	+	+	+	23	10	15	4	4	2	48	33	90	49	22.375	71.647
WBGene00000479	cgh-1	+			+	14	1					6		20	1	19.4	48.713
WBGene00012484	car-1	+			+	5						8	2	13	2	11.9	37.61
WBGene00000475	cey-4	+			+	4						3	1	7	1	18.35	32.418
WBGene00000474	cey-3	+			+	3						3	1	6	1	15.65	29.243
WBGene00000473	cey-2	+			+	6	2					7	3	13	5	34.8	29.404
WBGene00000105	alg-1	+			+	6	1					6	2	12	3	4.5	113.11
WBGene00021043	W05G11.6	+		+		9	3			4	2			13	5	12.95	73.199
WBGene00004435	rpl-23		+	+				4		2				6	0	27.15	14.954
WBGene00022235	sqd-1		+	+				3		2				5	0	10.7	33.673
WBGene00020696	T22F3.3		+	+				10	4	8	2			18	6	9.15	101.61
WBGene00015778	C14F11.1		+	+				3	1	3				6	1	10.15	45.617
WBGene00010556	rack-1			+	+					17	8	4	2	21	10	21.05	35.83
WBGene00001155	ech-6	+	+			2		3						5	0	13.05	31.172
WBGene00006423	asd-2				+							17		17	0	17.1	47.788
WBGene00006921	vha-12			+						15	4			15	4	23.8	54.75
WBGene00003776	nmy-1	+				11	2							11	2	5.8	229.37
WBGene00006789	unc-54	+				10								10	0	6.6	224.75
WBGene00002010	hsp-6	+				10	3							10	3	18.7	70.844
WBGene00001000	dim-1	+				9	1							9	1	14.5	71.849
WBGene00020347	T08B2.7			+						8	1			8	1	8.7	84.848
WBGene00004258	pyc-1			+						8	2			8	2	7.1	129.28
WBGene00004415	rpl-4			+						8	2			8	2	7	38.659
WBGene00001423	fib-1	+				7	1							7	1	16.5	36.383
WBGene00006840	unc-116	+				7	2							7	2	9.2	91.893
WBGene00006924	vig-1	+				6	1							6	1	26.2	40.406
WBGene00000381	cct-6	+				6	1							6	1	10.4	58.904
WBGene00009664	F43G9.1			+						6	1			6	1	15.6	38.466
WBGene00007150	B0365.1	+				5								5	0	6.1	120.6
WBGene00000140	anc-1	+				5								5	0	3.6	956.47
WBGene00002066	ifg-1	+				5								5	0	4.9	129.27
WBGene00017166	aldo-2	+				5	1							5	1	12.3	38.846
WBGene00004756	sec-24.2	+				5	1							5	1	4.7	109.34
WBGene00013340	Y59A8A.3	+				5	1							5	1	7.8	75.891
WBGene00004416	rpl-5		+					5						5	0	20.1	33.386
WBGene00012342	mtr-4	+				4								4	0	5.1	116.37
WBGene00004417	rpl-6	+				4								4	0	17.1	24.312
WBGene00004178	prg-1	+				4	1							4	1	5.6	93.843
WBGene00004447	rpl-33		+					4						4	0	25	13.768
WBGene00000217	asp-4	+				3								3	0	7.9	49.277
WBGene00020812	acdh-7	+				3								3	0	11.4	44.617
WBGene00008745	F13E6.1	+				3								3	0	15.4	21.287
WBGene00003903	pab-2	+				3								3	0	7.4	75.981
WBGene00006914	vha-5	+				3								3	0	3.9	99.312
WBGene00022456	Y110A7A.6	+				3								3	0	7.3	52.412
WBGene00001747	gsp-1	+				3								3	0	9.1	37.204
WBGene00015125	B0303.3	+				3								3	0	8	47.874
WBGene00002262	ldh-1			+						3				3	0	3.6	36.065
WBGene00000041	aco-2			+						3				3	0	5.8	84.046
WBGene00020511	immt-1				+							3		3	0	4.3	75.996

Figure S7

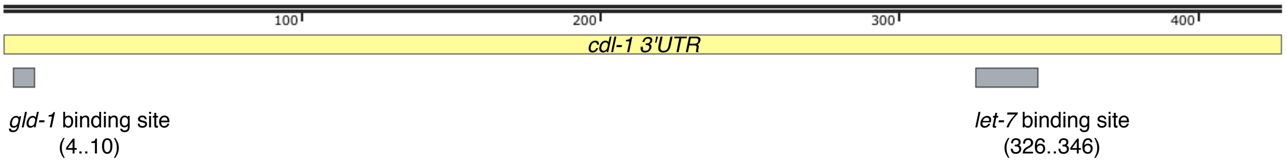

Figure Legends

Figure S1. *gld-1* genetically interacts with *nhl-2* and *vig-1* during germline development. (A) *nhl-2(ok818)* enhances sterility in *gld-1(op236)* animals. Synchronised animals were grown to adult stage and scored for the presence of stacked oocytes in at least one germ line in the indicated genetic backgrounds (error bars=95%C.I). (B) A representative picture of *gld-1(op236); nhl-2(ok818)* double mutant with stacked oocytes highlighted by the yellow line. The arrowhead indicates the spermatheca, which appears to be devoid of sperm. (C) Germline defects in *gld-1(op236); vig-1(ok2536).* DIC images of germlines reveal oocytes (indicated by yellow lines) in *gld-1(op236)* and *vig-1(ok2536)* mutants. *gld-1(op236); vig-1(ok2536)* double mutants are devoid of intact oocytes 36 h past L4 stage. (D) Egg laying rates in *gld-1(op236); vig-1(ok2536)*. Adult animals 24 h past L4 stage were placed on agar patches and allowed to lay eggs for 12 h in three 4-h intervals indicated as 4 h, 8 h and 12 h. Experiments were done in duplicate and five worms were included in each replicate. (E) Survival rates of *gld-1(op236); vig-1(ok2536)* embryos. Percentage survival indicates the number of eggs that hatched over the total number of eggs laid in the intervals described in (D) (error bars=S.D.)

Figure S2. *gld-1* doesn't affect *let-7* miRNA processing. Northern blot for *let-7* miRNA in different genetic backgrounds show no effect of *gld-1* on the mature miRNA levels. U6 snRNA is used as a control small RNA. Note the low *let-7* miRNA levels in *let-7(mg279)* as expected [1].

Figure S3. Somatic expression of a transcriptional *gld-1* reporter. DIC images (left panels), fluorescent images (middle panels) and merged images (right panels) of worms

expressing *pgld-1::mCherry-His::gld-1_3′UTR (gtEx2041)*. Cells expressing the transgene are indicated by white arrows in the middle panels.

Figure S4. Somatic expression of a rescuing operon transgene. Fluorescent images (left) and DIC images (right) of animals expressing the single copy insertion of the transgene [*gld-1p::GLD-1::gld-1 3′::operon linker::GFP::H2B::tbb-2 3′UTR]* in *gld-1(q485)* null mutants. GFP expression is seen in cells around the head region (top panel and middle panel) similar to the transcriptional GLD-1 reporter used in Figure S3. GFP expression is evident in all cells during embryonic development (bottom panel).

Figure S5. Changes in GFP levels in *let-7 sponge* strains. (A) Western blot of GFP levels in *let-7 sponge* and *let-7(mg279); let-7 sponge* worms. GFP levels are higher in *let-7(mg279)* worms. α-tubulin is used as a control. (B) Fold change in GFP levels detected by SILAC in *let-7(mg279); let-7 sponge* and *gld-1(op236); let-7(mg279); let-7 sponge* worms compared to the *let-7 sponge*. *let-7(mg279)* results in 1.44 fold increase in GFP that is under the control of *let-7* target *lin-41 3′UTR* (error bars=S.D).

Figure S6. Full list of protein interactors identified in GLD-1 immunoprecipitations. Highlighted in red are proteins that are linked to RNA regulation and / or miRNA pathway. Highlighted in blue are ribosomal related proteins. Yellow rows indicate proteins detected in both anti-GLD-1 Ab IP in wild type animals and in anti-GFP IP in GFP expressing animals. White rows are proteins detected either in anti-GLD-1 Ab IP in wild type animals or in anti-GFP IP in GFP expressing animals but not in both. For every protein, number of peptides detected in each IP together with peptides detected in control IPs (bc for background) are indicated. Total number of peptides detected and total number of background peptides are indicated in a separate column.

Figure S7. *cdl-1* 3′UTR with GLD-1 and *let-7* binding sites. *cdl-1* 3′UTR contains a single GLD-1 binding site [2] and a *let-7* binding site (mirWIP score 5.9, [3]) .

Movie S1. Movie depicting a lethargic *gld-1(op236); let-7(mg279)* worm

Table S1. Full list of proteins detected in SILAC experiments. 2179 proteins that passed our significance criteria described in materials and methods. Wormbase gene IDs (column A), log2 B/A ratios (column B), standard error of log2 B/A ratios (column C), B/A total peptides (column D), log2 C/A ratios (column E), C/A standard error (column F), C/A total peptides (column G), C/B fold change (column H), predicted as let-7 target in mirWIP database (column I), detected as GLD-1 target (column J). Where proteins are detected in two SILAC experiments standard errors are calculated. Standard errors of 0 indicate the proteins detected in one experiment only. * let-7 targets are obtained from the mirWIP database [3], and GLD-1 targets are obtained from [2,4].

References

1. Reinhart BJ, Slack FJ, Basson M, Pasquinelli AE, Bettinger JC, et al. (2000) The 21-nucleotide let-7 RNA regulates developmental timing in Caenorhabditis elegans. Nature 403: 901–906. doi:10.1038/35002607.
2. Wright JE, Gaidatzis D, Senften M, Farley BM, Westhof E, et al. (2011) A quantitative RNA code for mRNA target selection by the germline fate determinant GLD-1. EMBO J 30: 533–545. doi:10.1038/emboj.2010.334.
3. Hammell M, Long D, Zhang L, Lee A, Carmack CS, et al. (2008) mirWIP: microRNA target prediction based on microRNA-containing ribonucleoprotein-enriched transcripts. Nat Meth 5: 813–819. doi:10.1038/nmeth.1247.
4. Jungkamp A-C, Stoeckius M, Mecenas D, Grün D, Mastrobuoni G, et al. (2011) In Vivo and Transcriptome wide Identification of RNA Binding Protein Target Sites. Mol Cell 44: 828–840. doi:10.1016/j.molcel 2011.11.009.
